# Binding Mechanisms in Visual Perception and Their Link With Neural Oscillations: A Review of Evidence From tACS

**DOI:** 10.3389/fpsyg.2021.643677

**Published:** 2021-03-22

**Authors:** Andrea Ghiani, Marcello Maniglia, Luca Battaglini, David Melcher, Luca Ronconi

**Affiliations:** ^1^Department of General Psychology, University of Padua, Padua, Italy; ^2^Department of Psychology, University of California, Riverside, Riverside, CA, United States; ^3^Department of Neurobiology, University of Alabama at Birmingham, Birmingham, AL, United States; ^4^Neuro Vis.U.S. Laboratory, University of Padua, Padua, Italy; ^5^Department of Physics and Astronomy “Galileo Galilei”, University of Padua, Padua, Italy; ^6^Center for Mind/Brain Sciences and Department of Psychology and Cognitive Science, University of Trento, Trento, Italy; ^7^Psychology Program, Division of Science, New York University Abu Dhabi, Abu Dhabi, United Arab Emirates; ^8^School of Psychology, Vita-Salute San Raffaele University, Milan, Italy; ^9^Division of Neuroscience, Istituto di Ricovero e Cura a Carattere Scientifico, San Raffaele Hospital, Milan, Italy

**Keywords:** tACS, brain oscillations, temporal binding, spatial binding, feature binding

## Abstract

Neurophysiological studies in humans employing magneto- (MEG) and electro- (EEG) encephalography increasingly suggest that oscillatory rhythmic activity of the brain may be a core mechanism for binding sensory information across space, time, and object features to generate a unified perceptual representation. To distinguish whether oscillatory activity is causally related to binding processes or whether, on the contrary, it is a mere epiphenomenon, one possibility is to employ neuromodulatory techniques such as transcranial alternating current stimulation (tACS). tACS has seen a rising interest due to its ability to modulate brain oscillations in a frequency-dependent manner. In the present review, we critically summarize current tACS evidence for a causal role of oscillatory activity in spatial, temporal, and feature binding in the context of visual perception. For temporal binding, the emerging picture supports a causal link with the power and the frequency of occipital alpha rhythms (8–12 Hz); however, there is no consistent evidence on the causal role of the phase of occipital tACS. For feature binding, the only study available showed a modulation by occipital alpha tACS. The majority of studies that successfully modulated oscillatory activity and behavioral performance in spatial binding targeted parietal areas, with the main rhythms causally linked being the theta (~7 Hz) and beta (~18 Hz) frequency bands. On the other hand, spatio-temporal binding has been directly modulated by parieto-occipital gamma (~40–60 Hz) and alpha (10 Hz) tACS, suggesting a potential role of cross-frequency coupling when binding across space and time. Nonetheless, negative or partial results have also been observed, suggesting methodological limitations that should be addressed in future research. Overall, the emerging picture seems to support a causal role of brain oscillations in binding processes and, consequently, a certain degree of plasticity for shaping binding mechanisms in visual perception, which, if proved to have long lasting effects, can find applications in different clinical populations.

## Introduction

To build a representation of the incoming sensory flow, our brain needs to bind sensory information in a meaningful way, giving rise to a clear and smooth perception of the world. To do so, our visual system must integrate information across space and time and group together elements belonging to the same object. Magnetoencephalography (MEG) and electroencephalography (EEG) are high-temporal resolution techniques used to map brain dynamics, such as rhythmic patterns of neural activity that oscillates at a given frequency, known as brain oscillations. Brain oscillations have been linked to several cognitive and perceptual processes (Başar et al., 2001) and their synchronization has been suggested to modulate communication across long-range cortical regions (Fries, 2005, 2015; Bonnefond et al., 2017). Whether binding occurs in space (i.e., spatial binding) or time (i.e., temporal binding), M/EEG studies demonstrated a relationship between the power, phase, and synchronization of particular brain oscillations and binding processes (e.g., Varela et al., 1981; Rose and Büchel, 2005; Busch and VanRullen, 2010; Jensen et al., 2012; Hanslmayr et al., 2013; McLelland et al., 2016; Wutz et al., 2016; for reviews see VanRullen, 2016; Costa et al., 2017; Ronconi et al., 2017; White, 2018; Wutz et al., 2018). These electrophysiological studies provided us with valuable insights into the role of oscillatory activity in binding together efficiently sensory information to reach a meaningful representation. Indeed, integrating sensory information in space and time, like any other neurocomputational loop that implements effective communication between different cortical areas (e.g., top-down feedback from higher-order areas to lower-order sensory areas) would lead to the emergence of significant variations in the rhythmic oscillatory activity (Donner and Siegel, 2011; Siegel et al., 2012; Bastos et al., 2015; Jensen et al., 2015).

However, electrophysiological research is correlational in nature, meaning that it cannot demonstrate a causal role of the oscillatory activity in shaping our perception. To do so, we should directly manipulate brain oscillations and observe the effect on a behavioral level (for reviews see Herrmann et al., 2016; Bergmann and Hartwigsen, 2020).

A recent neuromodulation technique, transcranial alternating current stimulation (tACS), has been proven to be particularly suited for this goal (for reviews see Herrmann et al., 2013, 2016; Cabral-Calderin and Wilke, 2020; Kasten and Herrmann, 2020). Generally, tACS consists in the application of a weak sinusoidal electrical current on the scalp, through two rubber electrodes or through multi-channel devices (Dmochowski et al., 2011; Kuo et al., 2013). Several tACS parameters may be potentially manipulated: stimulation frequency, stimulation amplitude (that may range between 0.5 and 2 mA or adjusted according to participants thresholds) and finally, stimulation phase (Helfrich et al., 2014a,b; Strüber et al., 2014). There is increasing evidence that tACS is able to drive the endogenous rhythmic brain activity toward the chosen frequency of stimulation, transiently modulating brain oscillations in a frequency-dependent manner, a mechanism known as *entrainment* (Fröhlich and McCormick, 2010; Ozen et al., 2010; Herrmann et al., 2013; Reato et al., 2013). This mechanism has initially been supported by animal studies showing that the application of a sinusoidal current *in vitro* evoked spiking activity that synchronized with different external driving frequencies (Fröhlich and McCormick, 2010). Additionally, Ozen et al. found similar results by applying an extracranial sinusoidal current (Ozen et al., 2010). In humans, several studies supported entrainment by showing tACS effects on the EEG power spectrum during stimulation (Helfrich et al., 2014a,b) or after stimulation (Neuling et al., 2012; Battaglini et al., 2020a). However, other potential mechanisms of action have also been proposed. For example, Vossen et al. argued that tACS effects might be related to the modulation of spike timing-dependent plasticity, which would explain the offline effects of tACS (Vossen et al., 2015). Recent research also highlights the potential role of peripheral nerve or retinal stimulation in explaining tACS effects (Liu et al., 2018; Asamoah et al., 2019).

Liu et al. (2018) postulated five potential mechanisms of action to explain the immediate effects of stimulation on neural activity according to the applied electrical field intensity (i.e., the vector indicating the local change of the voltage as measured in Volts per meter; V/m). At the lowest electrical field intensities (i.e., <1 V/m) two mechanisms have been proposed: (1) *stochastic mechanism* and (2) *rhythmic mechanism*. In the first one a small proportion of the applied field would bias spike timing or probability of neurons that are near the threshold of spike generation thanks to the coincidence of intrinsic and extrinsic polarization. The second would take place when an endogenous rhythm is targeted with an alternating current of the same frequency, affecting the native oscillation at a similar phase during each cycle. At higher electrical field intensities, other mechanisms have been proposed, namely (3) *temporal bias of spikes*; (4) *entrainment* and (5) *imposed pattern*. The third mechanism is related to stochastic resonance (see point 1 above), but when the electrical field intensity is stronger neurons would be expected to be more reliably activated from trial to trial. Finally, when the difference between the external frequency and the endogenous rhythmic activity increases, a competition would arise and a higher electrical field would be needed in order to entrain less regular network patterns (entrainment) or to impose an arbitrary frequency on a neural network (imposed pattern) (Liu et al., 2018).

Another aspect worth mentioning is that brain stimulation effects may vary in a state-dependent manner (for reviews see Silvanto et al., 2008; Guerra et al., 2020). For example, it has been shown that alpha tACS can entrain brain oscillations when applied over the visual cortex, but only when the eyes were open (Ruhnau et al., 2016). Also, Feurra et al. showed that M1 excitability was differently affected by tACS stimulation according to participants' engagement in a motor task (i.e., resting condition vs. active observation condition) (Feurra et al., 2013). Intrinsic oscillatory dynamics is also important to consider, as tACS effectiveness likely depends on the proximity between the external stimulation frequency and the intrinsic rhythmic activity. For instance, alpha frequency strongly varies across subjects and the individual peak may be computed and used to properly set tACS stimulation frequency. Even if current findings support the idea that tACS can modulate brain oscillatory dynamics, future research should further investigate its precise physiological mechanisms to clarify the current debate over its underlying mechanism.

Even though its specific mechanism of action is still unclear, there is mounting evidence that tACS is able to modulate perceptually relevant brain oscillations. Additionally, brain oscillatory activity has been linked with temporal and spatial binding. Therefore, it is not surprising to observe a growing number of studies exploring the potential causal involvement of several brain rhythms in temporal and spatial binding. The evidence of a relationship between particular brain oscillations and perception, more generally, has been recently reviewed by Cabral-Calderin and Wilke (2020). Here, we constrained the scope of the review to focus specifically on different types of visual binding processes and their potential causal relationship with oscillatory activity.

The present review is structured into three main sections that focus on tACS studies exploring temporal, spatial, or spatio-temporal binding. After briefly introducing correlational evidence that motivates the tACS research, we describe each study and highlight its relevance in the understanding of the role of rhythmic activity in binding processes. Importantly, we highlight negative findings as well as methodological pitfalls and challenges that, if addressed, will improve reproducibility of neurostimulation research. We conclude by briefly discussing how tACS modulation of binding processes may be relevant for potential clinical interventions in clinical populations where these processes (and its oscillatory correlates) are disrupted.

## Temporal Binding

### Alpha Rhythm and Temporal Binding

It has been suggested that in order to integrate information over time, our brain may adopt a sampling mechanism that discretizes the sensory flow through discrete processing windows (Stroud, 1967). Several studies have suggested that this sampling mechanism is related to the endogenous alpha (8–12 Hz) activity (for reviews see VanRullen, 2016; White, 2018). This rhythm was shown to be linked to the temporal resolution of perception, both in the unimodal (Varela et al., 1981; Samaha and Postle, 2015; Milton and Pleydell-Pearce, 2016; Ronconi and Melcher, 2017; Ronconi et al., 2017, 2018; Wutz et al., 2018) and crossmodal domain (Grabot et al., 2017; Keil and Senkowski, 2017, 2018; Cooke et al., 2019; Bastiaansen et al., 2020; Migliorati et al., 2020), possibly by creating transient temporal windows of excitation and inhibition along its cycle that alternatively promote or reduce stimulus processing and effective communication between different cortical areas (Pöppel, 2009; Wutz and Melcher, 2014; VanRullen, 2016). Therefore, two stimuli will be integrated or segregated over time, depending on whether they fall within the same cycle or, in different oscillatory cycles. In other words, this intrinsic rhythmic process may be the means by which our brain could reach a balance between temporal integration and segregation of the sensory flow. Through temporal integration, sensory information is combined to reach an accurate representation of objects that remain stable over time. On the other hand, temporal segregation is needed when rapid changes must be detected, allowing for higher temporal resolution (i.e., higher sampling rate). However, most of these studies have established correlational evidence for the role of alpha activity, raising the question of whether this reflects a causal (sampling) mechanism or is merely an epiphenomenon.

**Figure 1:**
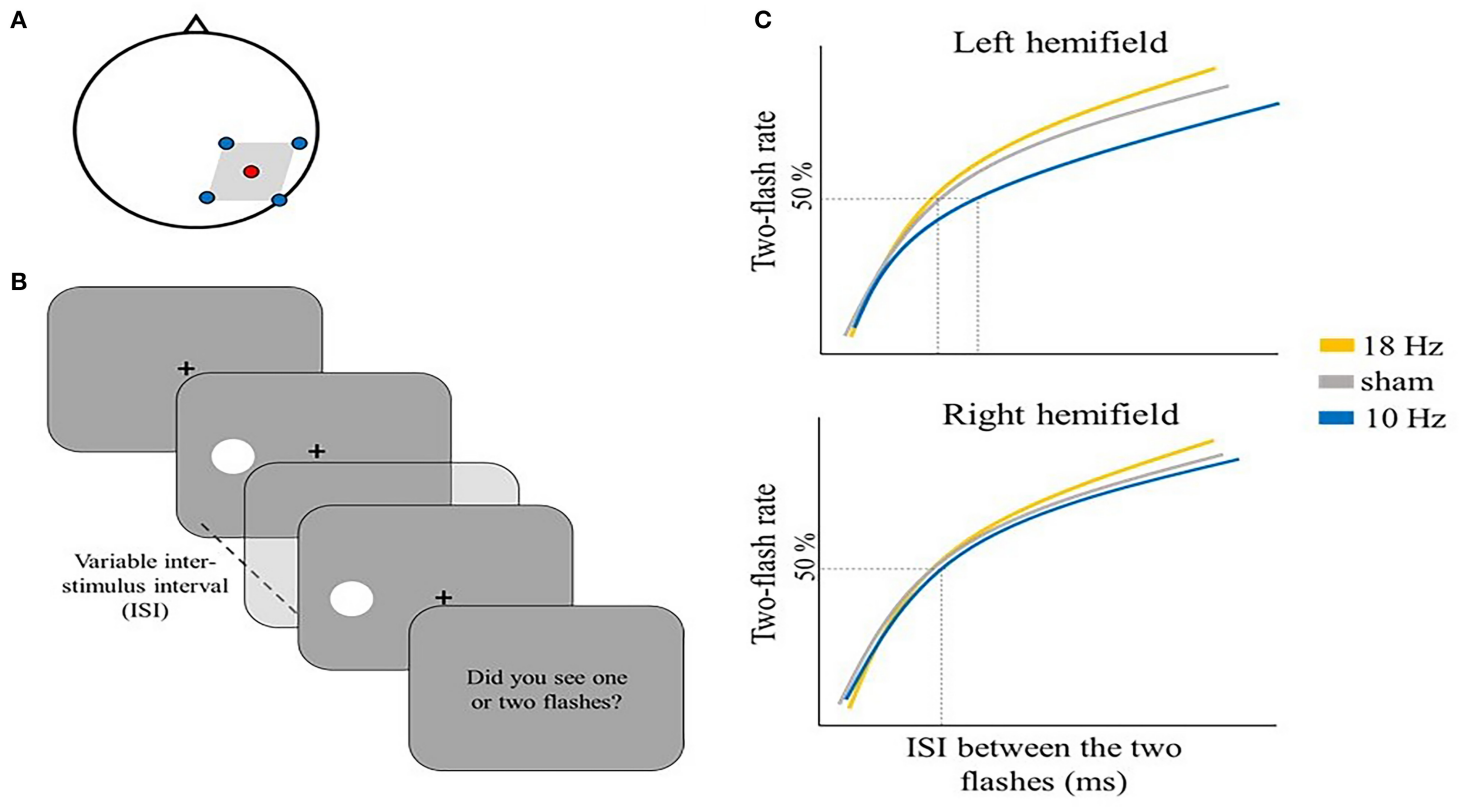
Illustrative representation of the study and results by Battaglini et al. (2020b). **(A)** mc-tACS montage: the stimulation electrode (red) was placed on PO8, the return electrodes (blue) were placed on Oz, P4, PO10, and TP8. **(B)** Schematic representation of one trial in the two-flash fusion task: two flashes were shown either on the left [as depicted in **(B)**] or right hemifield with a variable inter-stimulus interval ranging from 20 to 80 ms in steps of 10 ms. Participants were asked to report the number of perceived flashes. **(C)** Illustrative representation of the main results: the two-flash rate is shown as a function of the inter-stimulus interval between the two flashes for the three stimulation conditions, separately for the left hemifield (upper panel) and right hemifield (lower panel): 10 Hz tACS modulated the number of reported flashes, but only in the contralateral hemifield to stimulation (i.e., left hemifield).

Several studies have employed tACS to explore the potential causal involvement of alpha activity in the temporal resolution of perception. Battaglini et al. administered multi-channel (mc) tACS at 10 or 18 Hz and sham (i.e., placebo stimulation) over right V5/MT (electrode position PO8) (Figure 1A) to test selective effects of alpha tACS on two-flash fusion task performance.

Indeed, recent studies showed that (quasi) discrete temporal windows of integration, as measured by a two-flash fusion task, depend on the frequency (speed) (Samaha and Postle, 2015) and phase (Ronconi et al., 2017) of parieto-occipital EEG alpha activity.

In their task, participants were shown two sequential flashes, with an increase in the time interval between the flashes generally leading to a switch in perception from one to two unique stimulus events (Figure 1B).

Battaglini et al. (2020b) showed that participants tended to integrate two subsequent flashes more often (i.e., they tended to report just one flash) when 10 Hz tACS (i.e., alpha tACS) was applied, while 18 Hz tACS (i.e., beta tACS) and sham had no significant effect on the reported number of flashes. Also, the effect of right 10 Hz tACS was specifically found for stimuli in the contralateral visual hemifield (i.e., left) (Figure 1C). This is an important internal control as it excludes potential side effects of tACS like subthreshold retinal stimulation or skin sensations that would have been expected in both hemifields.

This study provided preliminary evidence of a causal role of alpha activity in the temporal resolution of perception. However, when comparing EEG recorded pre- and post-stimulation, the authors did not find any alpha power difference. This could indicate a lack of tACS effect or simply that the effect was short-lived. Moreover, the reported effects were found only when considering individual alpha frequency (IAF) as a covariate in their analysis. One important limitation is the application of a fixed stimulation frequency (i.e., 10 Hz) for all participants. Indeed, it has been shown that IAF (i.e., the frequency corresponding to the maximum power value in the EEG spectrum within the alpha band: Klimesch, 1999) may vary considerably across participants (Haegens et al., 2014). Therefore, a more sensitive approach would be to use each individual's alpha frequency (IAF) to select the tACS frequency (Zaehle et al., 2010). This approach should not only increase the effectiveness of stimulation (the entrainment is more likely to occur when the exogenous and endogenous frequencies are similar, Thut et al., 2011; Ali et al., 2013), but it would also provide new insights into the relationship between the IAF and the temporal resolution of perception, previously found to be associated (Samaha and Postle, 2015; Ronconi et al., 2018).

**Figure 2:**
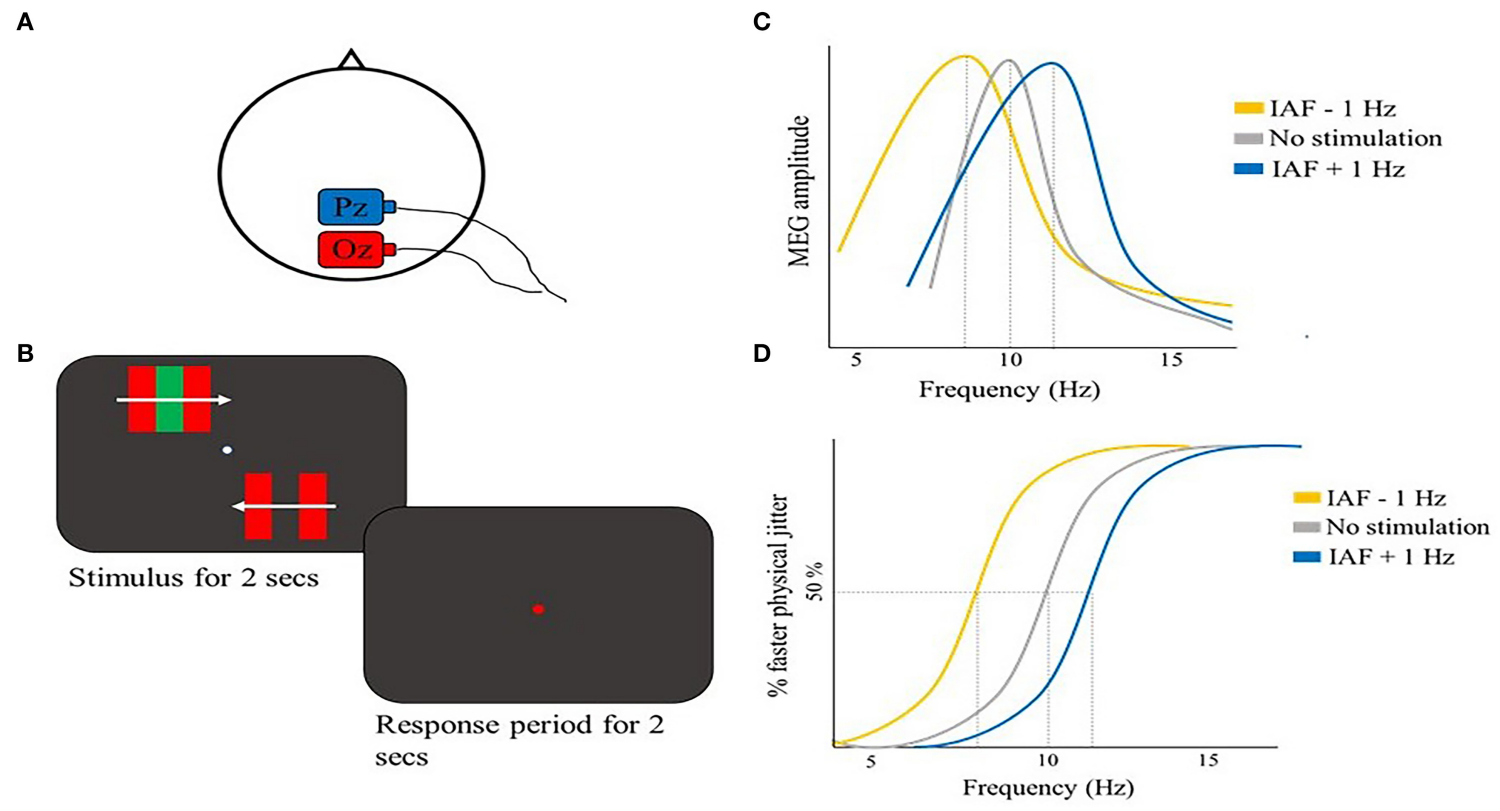
Illustrative representation of the study and results by Minami and Amano (2017). **(A)** tACS montage: the stimulation electrode (red) was placed on Oz, the return electrode (blue) was placed on Pz. **(B)** Schematic representation of the motion-induced spatial conflict task: participants reported whether the illusory jitter (upper visual field) was faster as compared to a physical jitter that could have 7 possible jitter frequencies (lower visual field). **(C)** Illustrative representation of the main results: MEG power spectrum of one participant is shown in the three stimulation conditions: IAF in the no stimulation condition was modulated in opposite directions according to tACS frequency (i.e., IAF −1 Hz or IAF + 1 Hz). **(D)** Psychometric function for the same participant in **(C)**. Perceived illusory jitter frequency changed depending on tACS stimulation frequency, congruently with IAF modulation.

A temporal binding study that took into account the IAF was performed by Minami and Amano (2017). The authors used a motion-induced spatial conflict task, where an illusory jitter is perceived when placing moving borders defined by color contrast near moving borders defined by luminance contrast (Arnold and Johnston, 2003). Participants were asked to judge whether a physical jitter on the lower visual field (whose frequency was randomly chosen among seven frequencies) was faster compared to an illusory jitter presented on the upper visual field (Figure 2B). First, they showed a correlation between the perceived illusory jitter frequency and the inter- and intra-participants variations of the individual peak alpha frequency (IAF), but no significant correlations were found with alpha power, beta frequency, or power, suggesting a specific role of alpha frequency in perceiving the illusory jitter. Crucially, in the same study, the authors showed that amplitude-modulated (AM) tACS (Witkowski et al., 2016) applied over parieto-occipital regions (electrode positions Pz and Oz) (Figure 2A) at IAF + 1 Hz not only enhanced IAF but also the perceived frequency of the illusory jitter. An opposite result was shown with tACS at IAF – 1 Hz (Figures 2C,D). It is noteworthy, however, that tACS IAF manipulation as revealed by MEG co-registration was effective only in 6 out of 12 participants. This finding suggests a high variability of tACS effectiveness across participants, likely due to impedance of the electrodes and/or skull thickness (Minami and Amano, 2017), highlighting the importance of inter-individual variations in assessing tACS efficacy. Still, the significant correlation between tACS-induced IAF changes and perceived jitter frequency strongly supports a causal role of alpha activity in modulating the temporal aspects of visual perception. In general, this study provided additional support for tACS as a tool to manipulate alpha oscillations at individually tailored frequencies, driving the endogenous oscillatory activity toward the external stimulation frequency.

**Figure 3:**
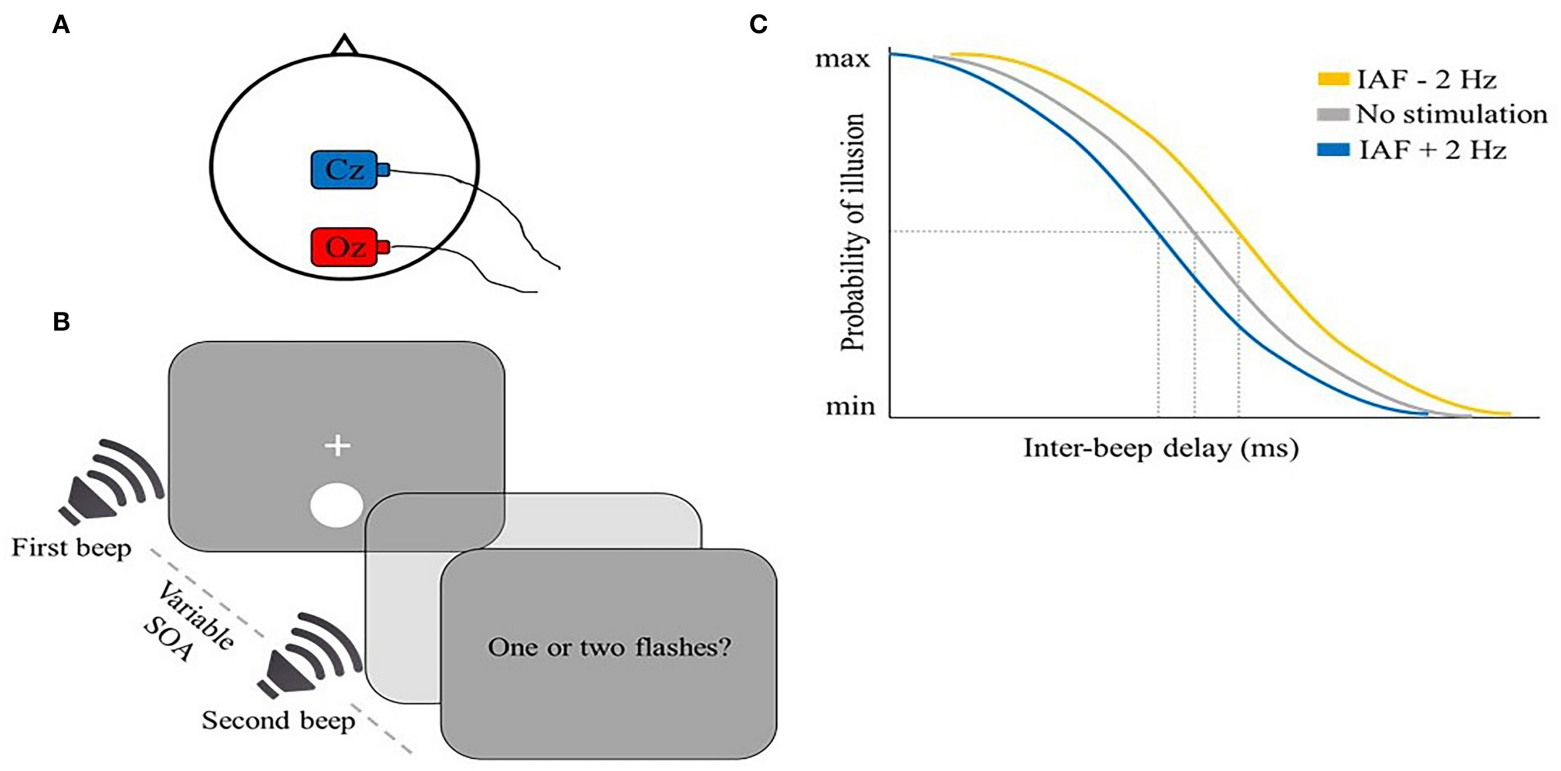
Illustrative representation of the study and results by Cecere et al. (2015). **(A)** tACS montage: the stimulation electrode (red) was placed on Oz, the return electrode (blue) was placed on Cz. **(B)** Schematic representation of the flash-beep task: a flash was shown together with a first beep, followed by a second beep after a variable time interval between 36 and 204 ms (12 ms steps). Participants were asked to report the number of perceived flashes. **(C)** Illustrative representation of the main results: the average perceived illusion is shown as a function of inter-beep delay for the three stimulation conditions: the size of the temporal window of illusion was modulated in opposite directions by higher (IAF + 2 Hz; blue) or lower (IAF – 2 Hz; yellow) tACS frequencies.

Some visual illusions induced by cross-modal interactions can be used as a probe to investigate the temporal sampling mechanisms of perception (Meredith et al., 1987; Slutsky and Recanzone, 2001; Cecere et al., 2015). For example, Cecere et al. (2015) explored a potential tACS modulation of the sound-induced double-flash illusion. In this illusion, when two beeps are shown together with one flash within 100 ms, a second illusory flash is usually perceived (Shams et al., 2000, 2002; Hirst et al., 2020) (Figure 3B). The authors found a correlation between the size of the temporal window of illusion and IAF, a result that suggests that alpha frequency may play a role in determining the temporal unit of integration. To directly test this hypothesis, in a second experiment the authors tried to modulate the size of the temporal window of illusion in opposite directions by applying tACS at IAF + 2 Hz and IAF – 2 Hz over the occipital cortex (electrode position Oz) (Figure 3A). As expected, when compared to IAF tACS, the two frequencies narrowed or broadened this window, respectively (Figure 3C). This finding was proposed to reflect a direct IAF modulation toward the stimulation frequency. However, this conclusion remains speculative, as no M/EEG signal was recorded during or after tACS application.

**Figure 4:**
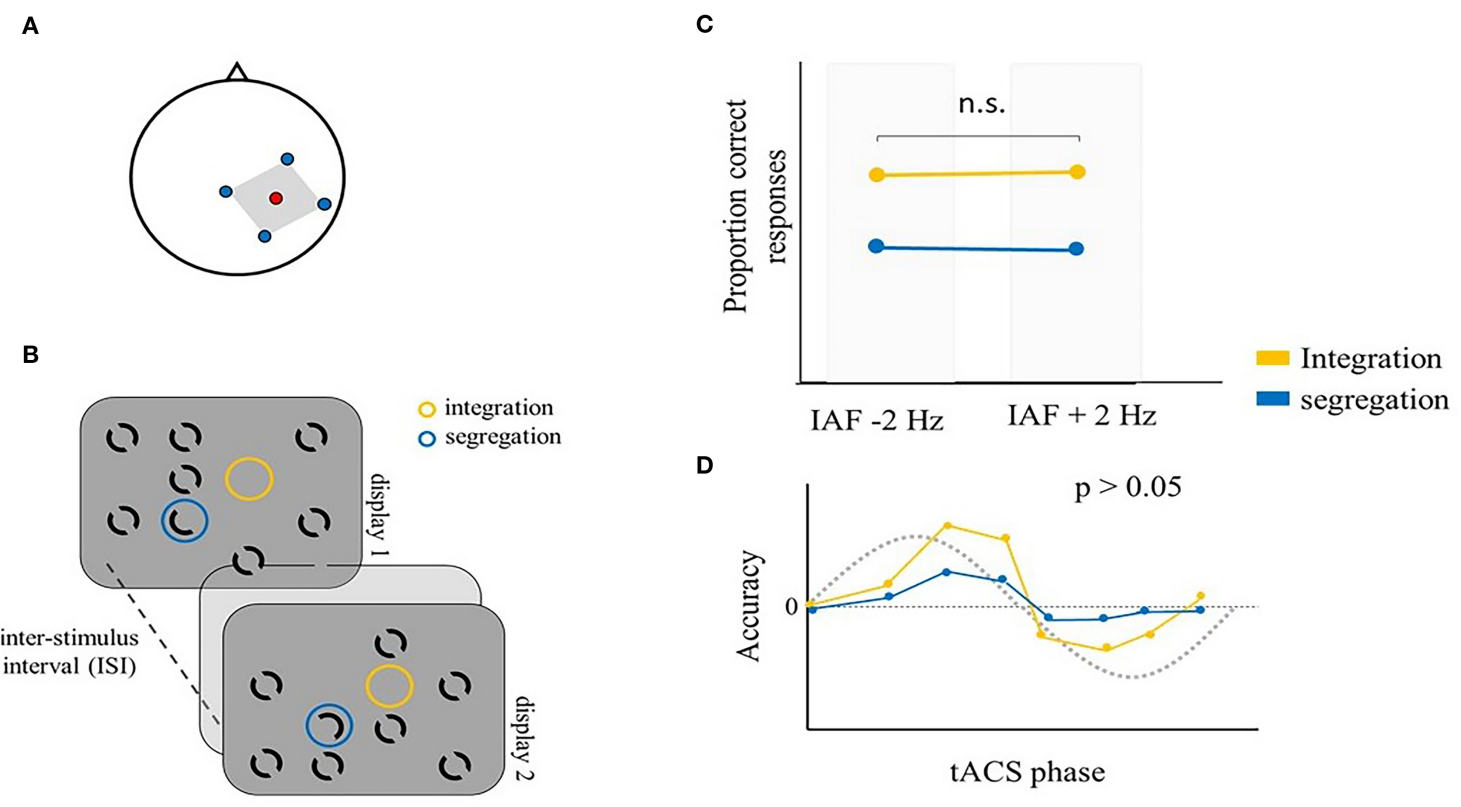
Illustrative representation of the study and results by Ronconi et al. (2020a). **(A)** mc-tACS montage: the stimulation electrode (red) was placed on P4, the return electrodes (blue) were placed on C4, Pz, O2, and P8. **(B)** Schematic representation of the SegInt task: two displays were successively shown, separated by a blank interval. Both displays presented seven full black annuli, an “odd element” (blue circle), with just half annulus in each display and an empty location (yellow circle) in both displays. Participants were asked to indicate either the position of the odd element (segregation) or the empty location (integration). **(C)** The proportion of correct responses in integration and segregation trials was not significantly different between IAF – 2 Hz tACS and IAF + 2 Hz tACS. **(D)** Temporal integration and segregation accuracy as a function of tACS phase bin. The best fitting sinusoidal function was not significant, suggesting that in this study, tACS was not able to reliably modulate segregation and integration processes in a sinusoidal fashion.

These studies provide evidence that tACS modulates temporal binding processes in the visual domain in a frequency-dependent manner. They also suggest that tACS effectiveness can be increased by custom tailoring the stimulation frequency individually for each participant. However, other studies did not show such modulation. Ronconi et al. (2020a) employed mc-tACS over the right parietal cortex (electrode position P4) (Figure 4A) at individually tailored alpha frequencies (IAF ± 2 Hz) to test visual temporal segregation and integration. Indeed, as previously reported, a better segregation performance is expected with higher alpha frequencies (e.g., IAF + 2 Hz), while an improved integration should be associated with lower alpha frequencies (e.g., IAF – 2 Hz) (Ronconi and Melcher, 2017; Ronconi et al., 2018). They used an alternative version of the missing-dot task (Eriksen and Collins, 1967), the SegInt task, which measures temporal segregation and integration performance (Wutz et al., 2016, 2018; Ronconi et al., 2018; Sharp et al., 2019). In this task, two displays are shown in sequence, separated by a blank interval. Both displays present seven black annuli, one “odd element” composed by a half annulus, and an empty location (Figure 4B). Participants were alternatively asked to indicate the position of either the odd element or the empty location, allowing to measure integration and segregation performance with the same stimuli, but different instructions, in separate blocks. Indeed, segregation of the two displays is needed to perceive the odd element, while their integration is required to perceive a unique empty location. Previous work had employed sensory entrainment to modulate performance in the SegInt task (Ronconi et al., 2018). In contrast, they reported no modulation of alpha frequency by mc-tACS, as measured with EEG immediately after parietal mc-tACS, nor a variation of segregation and integration performance during the stimulation (Figure 4C). Even if other studies succeeded in showing modulation of temporal aspects of perception with alpha tACS (Cecere et al., 2015; Minami and Amano, 2017; Battaglini et al., 2020b), the study by Ronconi et al. highlights important limitations of tACS research that should be addressed in future studies, namely, electrode montage and electric current orientation, the selection of the stimulation frequency and the differential effects of stimulation at the anodes and cathodes. The consideration of all these aspects would ideally involve computational models, electric field modeling, and individualized dose-controlling in order to increase the reproducibility of neurostimulation research (Wagner et al., 2014; Bikson et al., 2018; Evans et al., 2020). This study also attempted to investigate a possible phase-dependent alpha tACS modulation of integration/segregation accuracy. Their hypotheses were driven by evidence that, in addition to frequency, the phase of endogenous oscillations has been shown to play a modulatory role in perception (Busch et al., 2009; Mathewson et al., 2009; Busch and VanRullen, 2010; Neuling et al., 2012). Therefore, within a temporal binding task, two subsequent stimuli are temporally bound together depending on whether they are presented within preferential phase points along the oscillatory cycle. If tACS is effectively able to induce entrainment, then we would expect the endogenous activity to be in-phase with the external current after entrainment has occurred. This means that if the behavioral performance oscillates periodically according to the phase of the ongoing cycle, tACS stimulation should lead to a sinusoidal modulation of any measure of task performance. Support for this view comes from studies that showed a phase-dependent modulation of occipital alpha tACS in target detection accuracy (Helfrich et al., 2014b) and reaction times in three visual tasks (de Graaf et al., 2020). To explore a possible phase-dependent tACS modulation, Ronconi et al. (2020a) computed the exact phase point at the onset of each trial and divided the tACS alpha cycle into eight bins. By comparing performance across these phase bins, they were able to explore whether a phase-specific modulation of performance emerged from their data. Even if they found preliminary evidence of a relation between integration accuracy and tACS phase, a more stringent permutation test did not confirm the strength of the effect (Figure 4D) (Ronconi et al., 2020a). In sum, even if tACS was previously shown to shape detection accuracy in a sinusoidal fashion (Helfrich et al., 2014b; de Graaf et al., 2020), this study provides only partial evidence on whether tACS is able to effectively modulate segregation/integration performance in a phase-dependent manner.

### Other Rhythms Involved in Temporal Binding

So far, we mainly focused on studies that investigated the role of alpha activity in shaping the temporal resolution of perception, as this is one of the main rhythms that was first suggested by electrophysiological studies (Busch and VanRullen, 2010; Milton and Pleydell-Pearce, 2016; VanRullen, 2016; Ronconi et al., 2018). However, it should be noted that other rhythms were found to be associated with transient temporal windows of processing, like the theta rhythm (Busch et al., 2009; Ronconi et al., 2017). For example, Ronconi and Melcher found a phase-dependent modulation of segregation/integration performance after sensory entrainment in the alpha and theta frequency, suggesting a role of both frequencies in segregating/integrating stimuli across the temporal domain (Ronconi and Melcher, 2017). Given this evidence, one may expect to find tACS studies that aimed to modulate theta activity to investigate its potential causal role in temporal binding. In principle, the same logic applied to alpha frequency in previous studies may be applied to theta frequency as well. However, even if several studies investigated the role of theta activity in other cognitive domains with tACS (Polanía et al., 2012; Pahor and Jaušovec, 2014; Vosskuhl et al., 2015; Chander et al., 2016; Wischnewski et al., 2016), to the best of our knowledge, there are currently no tACS studies focusing on the causal relationship between theta activity and temporal binding windows.

Temporal integration/segregation processes have also been related to the attentional blink (Shapiro et al., 1997; Giesbrecht et al., 2004; Kim and Blake, 2005). The attentional blink (AB) occurs when a second target (T2) embedded in a rapid serial visual presentation is missed when following a first target (T1) within a time interval between 200 and 500 ms (Raymond et al., 1992). Interestingly, T2 detection performance is often spared when it appears at the so-called Lag 1 position (i.e., right after T1). Several theories tried to explain the phenomenon of Lag1 sparing. The theory proposed by Akyürek and Hommel (2005) claims that performance at Lag 1 during an AB task may be accounted for by temporal integration of T1 and T2 into a single percept, meaning that performance at Lag1 may be considered as a measure of temporal integration. Additionally, EEG studies showed that correct T2 detection was associated with an increase in beta-band coherence and decrease in alpha-band coherence between frontal and parietal regions (Kranczioch et al., 2007; Janson and Kranczioch, 2011) or with variations in pre-stimulus alpha power (MacLean and Arnell, 2011; Ronconi et al., 2016a), meaning that within an AB task, the conscious perception of subsequent rapid stimuli likely depends on alpha and beta oscillations (Shapiro et al., 2017). A potential tACS modulation of T2 detection performance at Lag 1 would add support to a causal role of fronto-parietal beta and alpha synchronization in resolving correct segregation of T1 and T2 in an AB task. To this aim, Yaple and Vakhrushev (2018) employed a standardized AB paradigm (Shapiro et al., 1994) and applied 10 Hz (i.e., alpha) or 20 Hz (i.e., beta) tACS targeting both right parietal (electrode position P4) and left frontal (electrode position F3) cortices either with an in-phase or out-of-phase stimulation. Given the oscillatory correlates related to a successful T2 detection (Kranczioch et al., 2007), they expected to observe an increase in T2 detection accuracy during an in-phase beta stimulation and out-of-phase alpha stimulation. As expected, they found an increased performance at Lag 1. Surprisingly, they also found an improved T2 detection after out-of-phase 20 Hz tACS and no effect after alpha stimulation. Critically, however, they reported a non-significant interaction between stimulation condition and Lag, meaning that different tACS stimulations do not differently affect task performance according to the Lag levels (e.g., Lag 1), as we would have expected considering performance at Lag 1 as an index of temporal integration.

### Temporal Binding: Summary

To summarize, in this section we described studies investigating the relationship between temporal binding processes and oscillatory activity with tACS (see Table 1 for a summary of these studies). Overall, the findings summarized above support a causal role of brain oscillations in shaping the temporal resolution of perception. There is mounting evidence that tACS is able to selectively modulate EEG alpha activity, a rhythm previously shown to correlate with temporal binding processes. In particular, there is increasing evidence in favor of a selective tACS modulation of alpha frequency when applied to occipital areas at faster or slower frequencies compared to the individual alpha frequency (Cecere et al., 2015; Minami and Amano, 2017). This is in line with previous electrophysiological research showing a link between temporal binding and IAF (Samaha and Postle, 2015; Ronconi et al., 2018). However, a recent study which applied alpha tACS at a fixed stimulation frequency (i.e., 10 Hz) still found an effect on participants' temporal binding performance (Battaglini et al., 2020b). This may be due to the proximity between the target tACS frequency (10 Hz) and the average endogenous alpha frequency. In fact, as reported in this study, the average IAF was very close to 10 Hz and the majority of the computed IAFs fell within 10 Hz ± 1. Moreover, the stimulation effect was significant only when considering IAF as a covariate, meaning that tACS effect depended on IAF values, as shown by previous studies.

**Table 1:** Summary of tACS studies investigating the causal role of brain oscillations in binding mechanisms.

	**References**	**tACS**	**Electrodes position**	**Stimulation parameters**	**M/EEG registration**	**Experimental Paradigm**	**Behavioral outcome**	**M/EEG outcome**
Temporal binding	Battaglini et al. (2020b)	mc-tACS StarStim8, NIC No phosphenes reported	1 × 4 channels: PO8 (anode)-Oz, P4, PO10, TP8 (cathodes) in 10–10 system. For all electrodes: 2 cm diameter	Target frequency: 10 Hz Active control: 18 Hz Sham: present Intensity/duration: 1.6mA for 45 min	EEG pre- and post-tACS	Two-flash fusion task	10 Hz tACS reduced the temporal resolution of perception	10 Hz tACS did not selectively increase alpha power after stimulation
	Minami and Amano (2017)	AM-tACS, NeuroConn No phosphenes reported	Pz-Oz (5 × 7 cm^2^) in 10–20 system	Target frequency: IAF ± 1 Hz Active control: none Sham: present Intensity/duration: 2 mA for 56 s per block	MEG pre- and during tACS	Motion-induced spatial conflict	IAF + 1 Hz and IAF – 1 Hz modulated the perceived jitter frequency	AM-tACS successfully manipulated PAF
	Cecere et al. (2015)	Standard tACS Magstim, UK Intensity decreased until no phosphenes were reported	Oz (anode,9 cm^2^)-Cz (cathode, 35 cm^2^) in 10–20 system	Target frequency: IAF ± 2 Hz Active control: IAF Sham: absent Intensity/duration: 2 mA for 10 min	EEG pre-tACS	Flash-beep task (sound-induced double flash illusion)	Compared to IAF tACS, IAF+2 Hz tACS (IAF– 2 Hz) narrowed (broadened) the temporal window of the sound induced double flash illusion	No M/EEG during or after stimulation
	Ronconi et al. (2020a)	mc-tACS StarStim 8, NIC No phosphenes reported	1 × 4 channels: P4 (anode)-C4, Pz, O2, P8 (cathode) in 10–10 system. For all electrodes: 2 cm diameter	Target frequency: IAF ± 2 Hz Active control: none Sham: absent Intensity/duration: 2 mA for 40 min	EEG pre- and post-tACS	Segregation/integration task	IAF ± 2 Hz mc-tACS did not modulate integration/segregation performance, as expected	IAF ± 2 Hz mc-tACS did not modulate endogenous IAF
Spatial binding	Stonkus et al. (2016)	4 channel DC Stimulator, NeuroConn	PO7, CP6 (anodes, diameter 3.7 cm)—POz (cathode, diameter 3.7 cm) in 10–20 system	Target frequency: 7 Hz (in-phase or out-of-phase) Active control: none Sham: present Intensity/duration: 2 mA for 1,000–1,800 ms pre-stimulus	EEG post-tACS	Contour integration task	In-phase stimulation improved contour detection performance. However, anti-phase stimulation did not show any effect	Prestimulus tACS caused entrainment echoes
	Battaglini et al. (2020a)	mc-tACS StarStim8, NIC No phosphenes reported	1 × 4 channels: P4 (anode)-C4, Pz, O2, P8 (cathodes) in 10–10 system. For all electrodes: 2 cm diameter	Target frequency: 18 Hz Active control: 10 Hz Sham: present Intensity/duration: 1.6 mA for 45 min	EEG pre- and post-tACS	Letter orientation discrimination task	18 Hz tACS improved performance in a crowded condition No tACS phase modulation was found	18 Hz tACS selectively increased beta power after stimulation
Feature binding	Zhang et al. (2019)	DC stimulator, NeuroConn intensity was decreased until no phosphenes were reported	Main experiment: PO3 (anode)-Cz (cathode) Control: PO4 (anode) -Cz (cathode) (all electrodes: 35 cm^2^) in 10–20 system	Target frequency: IAF (exp 1); IAF ± 2 Hz (exp 2) Active control: IAF (exp 2) Sham: present (exp 1-2) Intensity/duration: 2 mA for 18 min	EEG pre-tACS	Bistable color-motion binding task	IAF tACS reduced the time proportion of active (illusory) binding. IAF+2 Hz and IAF – 2 Hz tACS selectively enhanced and reduced perceptual switch rate	No M/EEG during or after stimulation
Spatio-temporal Binding	Strüber et al. (2014)	DC stimulator, NeuroConn No phosphenes reported	Experiment 1–2 (out-of-phase): P7-PO7 (5 x 7 cm) and P8-PO8 (5 × 7 cm) in 10–10 system Experiment 3 (in-phase): dual-channel O1 (anode)-C3 (cathode), O2 (anode)-C4 (cathode)	Target frequency: 40 Hz (in-phase or out-of-phase) Active control: 6 Hz (in-phase or out-of-phase) Sham: present Intensity/duration: individually adjusted intensity (in mA) for 15 min	EEG pre- and post-tACS	Stroboscopic alternative motion paradigm	Anti-phase 40 Hz tACS decreased the proportion of perceived horizontal motion	Anti-phase 40 Hz tACS increased interhemispheric gamma coherence
	Helfrich et al. (2014a)	mc-tACS, DC stimulator Plus NeuroConn No phosphenes reported	1 × 4 channel tACS on left and right extra-striate visual cortex	Target frequency: 40 Hz (in-phase or out-of-phase) Active control: none Sham: present Intensity/duration: 1 mA for 20 min	EEG pre- during and post-tACS	Stroboscopic alternative motion paradigm	40 Hz in-phase (out-of-phase) stimulation promoted the perception of the horizontal motion (vertical motion)	In-phase and out-of-phase 40 Hz tACS modulated gamma interhemispheric synchrony, but not gamma power. 40 Hz tACS decreased alpha power
	Kar and Krekelberg (2014)	Standard tACS STG4002	PO7-PO3 (anode)-Cz (cathode) in 10–20 system. For all electrodes: 7.60 cm diameter	Target frequency: 10 Hz Active control: none Sham: absent Intensity/duration: 1.0 mA for 40 s or 4 s	No M/EEG	Motion discrimination task	10 Hz tACS improved motion discrimination sensitivity likely by reducing motion adaptation	No M/EEG during or after stimulation
	Cabral-Calderin et al. (2015)	DC stimulator Plus, NeuroConn	Oz (anode, 16 cm^2^)-Cz (cathode, 35 cm^2^) in 10-20 system	Target frequency: 10 Hz; 60 Hz; 80 Hz Active control: none Sham: present Intensity/duration: 1.5 mA for 5 min	No M/EEG	Structure-from-Motion stimulus	60 Hz tACS, but not 10 Hz tACS, increased the perceptual switch rate, promoting perceptual reorganization during ambiguous stimulation	No M/EEG during or after stimulation

*The reported tACS amplitude in Stimulation parameters is peak-to-peak. AM, amplitude-modulated; mc, multi-channels; tACS, transcranial alternating current stimulation; IAF, individual alpha frequency; mA, milliAmpere; μA, microAmpere; PAF, peak alpha frequency*.

In line with the electrophysiological literature (for reviews see VanRullen, 2016; White, 2018), some studies also showed a phase-dependent modulation of accuracy and reaction times induced by occipital tACS in detection tasks (Helfrich et al., 2014b; de Graaf et al., 2020). Nonetheless, it should be noted that other studies using parietal tACS did not convincingly find consistent evidence in a task that directly measured both integration and segregation performance (Ronconi et al., 2020a). This study addressed possible issues concerning tACS effectiveness such as the choice of the stimulation montage and current distribution modeling that should be investigated more deeply in future studies to increase tACS efficacy in the exploration of visual temporal binding. Finally, another aspect that is worth mentioning is that no tACS studies to date explored the role of theta activity in temporal binding processes. Considering the increasing number of M/EEG studies showing a role of this rhythm in the temporal dimension of perception (Busch et al., 2009; Wutz et al., 2016; Ronconi et al., 2017) and the fact that theta tACS was successfully applied in the investigation of other cognitive domains (Polanía et al., 2012; Pahor and Jaušovec, 2014; Vosskuhl et al., 2015; Chander et al., 2016; Wischnewski et al., 2016), future research should be guided by such correlational evidence and begin to explore the role of multiple oscillatory rhythms in shaping perception in the temporal domain.

## Spatial Binding

Extensive literature shows that, as for temporal binding, brain oscillations are related to binding processes within the spatial domain as well (Hanslmayr et al., 2013; Costa et al., 2017). In order to solve the “binding problem” (e.g., which features belong to which object) our visual system must dictate a meaningful structure that emerges from grouping together elements belonging to the same object or spatial location (Treisman, 1998). Even within a sensory modality (e.g., vision), there are specialized areas for the processing of particular features (e.g., color, shape, size, motion: Livingstone and Hubel, 1988). This poses the problem of how object features are integrated together and segregated from features belonging to other objects (Varela et al., 2001; Robertson, 2003). One potential mechanism is the *temporal binding hypothesis* (Singer and Gray, 1995), according to which features that are integrated together are encoded by synchronous neural activity. Several findings support this view by showing synchronous neural activity among distant neural networks during bindings within the visual system (Rose and Büchel, 2005; Hipp et al., 2011). However, other studies did not find any relationship between synchrony and perceptual grouping (Thiele and Stoner, 2003; Palanca and DeAngelis, 2005). Later, Roelfsema proposed another possible account, the *incremental grouping theory*, where neurons encoding the features of one object increase their firing rate compared to neurons encoding the features of another object (Roelfsema et al., 2004; Roelfsema, 2006). Irrespective of the framework, there are computational and theoretical reasons to suggest that spatial grouping is likely achieved by an interplay between feedforward and feedback activity within the visual system (Roelfsema, 2006; Jehee et al., 2007). While the feedforward connections would promote the representation of visual features within a spatial map (Tootell et al., 1998), feedback activity from higher-level regions would promote the selection of targets according to their spatial location (Foxe and Snyder, 2011).

It has been suggested that, in order to solve a contour integration problem, information is bidirectionally exchanged between occipital and parietal regions, namely, between left LO1 and right intraparietal sulcus (Hanslmayr et al., 2013). While LO1 would preferentially respond to orientation (Larsson and Heeger, 2006) and collinearity (Altmann et al., 2003; Kourtzi et al., 2003) of local elements, synchronization of this region with the parietal cortex would provide a spatial reference by enhancing LO1 neurons firing rates in the relevant locations through incremental grouping (Roelfsema, 2006), promoting the integration of local elements across space. Theta prestimulus phase has been shown to predict this kind of visual perceptual binding, suggesting that theta rhythm may transiently regulate gating of information within a contour integration task, likely by opening time windows for information exchange between low-level occipital regions and high-level parietal regions (Hanslmayr et al., 2013). Indeed, functional connectivity between left occipital and right parietal regions in Hanslmayr et al. (2013) was modulated according to the phase of ongoing theta oscillations, such that no significant connectivity between parietal and lateral occipital areas was evident at a certain angle of EEG theta phase (i.e., least optimal phase angle), while strong connectivity was shown at opposite phases (i.e., optimal phase angle). Starting from this correlational evidence, Stonkus et al. tested a causal role of these theta oscillations in a similar contour integration task (Stonkus et al., 2016). In order to investigate the causal role of pre-stimulus theta frequency, tACS was applied only during the pre-stimulus period (i.e., fixation). Participants asked to indicate whether a contour formed by aligned oriented Gabor stimuli was shown among other randomly oriented Gabor patches. Electrodes were placed to optimally stimulate the left middle occipital cortex (electrode position PO7) and the right inferior parietal lobule (electrode position CP6). The two key stimulation conditions were the in-phase and out-of-phase 7 Hz (i.e., theta) tACS delivered to these two regions. Results showed that participants were more accurate in integrating and detecting the contour when in-phase stimulation was delivered. However, out-of-phase stimulation did not impair performance, as it would have been expected if the putative oscillatory correlate was causally involved in contour integration. Critically, they reported a 7 Hz tACS phase effect on performance by showing a sinusoidal modulation in the in-phase condition. Moreover, to test possible neurophysiological effects of prestimulus tACS, post-stimulation EEG activity phase-locked to tACS offset was recorded, showing the presence of entrainment echoes (i.e., short-lived oscillatory aftereffects phase-locked to the offset of the rhythmical stimulation). This adds support to the idea that tACS can entrain neural oscillations in a time-sensitive manner (Stonkus et al., 2016). Even if the same authors recommended caution in the interpretation of these results due to some methodological limitations (e.g., tACS was not continuous, but applied only for a brief period before stimulus presentation, fixed stimulation frequency, no effect of out-of-phase stimulation, massive contamination of the EEG signal), these findings partially support the causal role of theta activity in contour integration and importantly, they provide additional evidence for the use of tACS as a tool to shape brain oscillations relevant in binding processes even when applied in a brief pre-stimulus interval.

**Figure 5:**
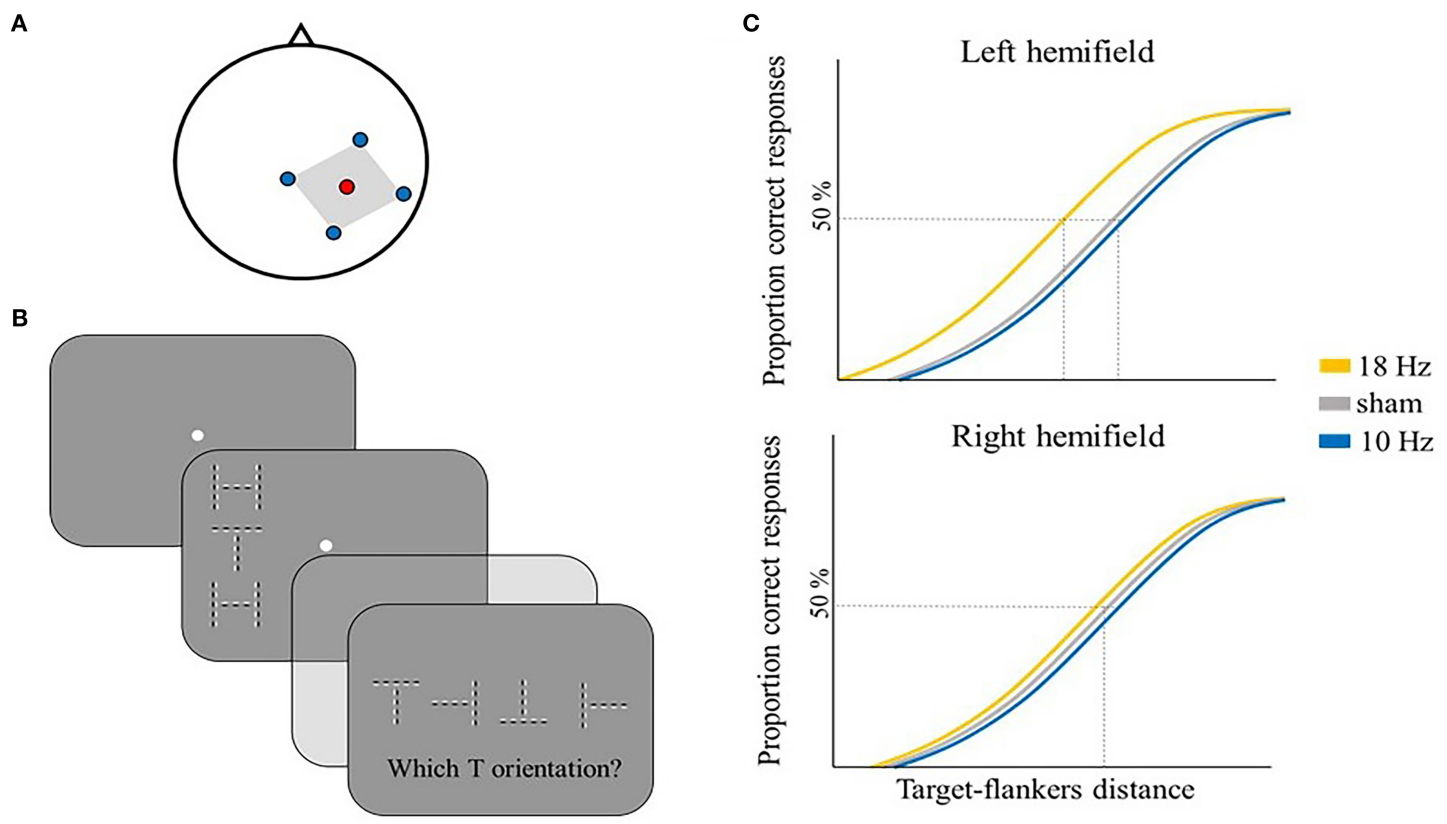
Illustrative representation of the study and results by Battaglini et al. (2020a). **(A)** mc-tACS montage: the stimulation electrode (red) was placed on P4, the return electrodes (blue) were placed on C4, Pz, O2, and P8. **(B)** Schematic representation of one trial in the letter orientation discrimination task. H letters (i.e., flankers) could have 7 possible distances from the T letter (i.e., target), creating different levels of visual crowding. Participants were asked to indicate the T orientation in each trial. **(C)** Illustrative representation of the main results: the proportion of correct responses is shown as a function of the target-flankers distance for the three stimulation conditions, separately for the left hemifield (upper panel) and right hemifield (lower panel): 18 Hz tACS (yellow) increased participants performance, but only in the contralateral hemifield to stimulation (i.e., left hemifield).

Another study, Battaglini et al. (2020a), investigated the relationship between brain oscillations and spatial integration in conditions of visual crowding, where the detectability of a target is impaired by the presence of flanking elements. Previous studies showed a relationship between beta-band activity and task performance in visual crowding, such that higher beta amplitude correlates to increased performance within a crowding regime (Ronconi et al., 2016b; Ronconi and Bellacosa Marotti, 2017). In order to test the causal role of beta activity in resolving correct target feature integration in a crowded condition, Battaglini and colleagues (Battaglini et al., 2020a) applied sham, 10 Hz (i.e., alpha) tACS, and 18 Hz (i.e., beta) tACS over the right parietal cortex (electrode position P4) (Figure 5A). They used a letter orientation discrimination task varying the critical space between the target (T letter) and two vertically positioned flankers (H letters) (Figure 5B). Participants were instructed to report the T orientation presented in each trial. Results showed improved performance during beta tACS as compared to alpha tACS and sham (Figure 5C). This finding was explained in terms of a selective role of beta oscillations in facilitating dorsal-to-ventral feedback which has been suggested to promote a switch from global (crowded) to local (uncrowded) visual processing by activating appropriate integrative filters for detail-oriented visual processing (Hochstein and Ahissar, 2002; Jehee et al., 2007). This study also explored a possible tACS phase modulation of performance. As described previously, a sinusoidal modulation of task performance should be expected if we assume entrainment to be generated by tACS stimulation. Even if Battaglini et al. (2020a) found a selective enhancement of EEG beta power after beta tACS (i.e., tACS was effective in modulating beta power after stimulation), they reported no sinusoidal modulation of task performance, as it would have expected by genuine entrainment. Thus, even though alpha and theta tACS have been shown to modulate performance depending on the specific stimulation phase (Helfrich et al., 2014b; Stonkus et al., 2016, de Graaf et al., 2020), there is no evidence at present that the logic of “duty cycle” applied to lower oscillatory bands activity (e.g., theta and alpha) could be also valid for higher oscillations (e.g., beta oscillations) (Samaha et al., 2017; Battaglini et al., 2020a).

## Feature Binding

**Figure 6:**
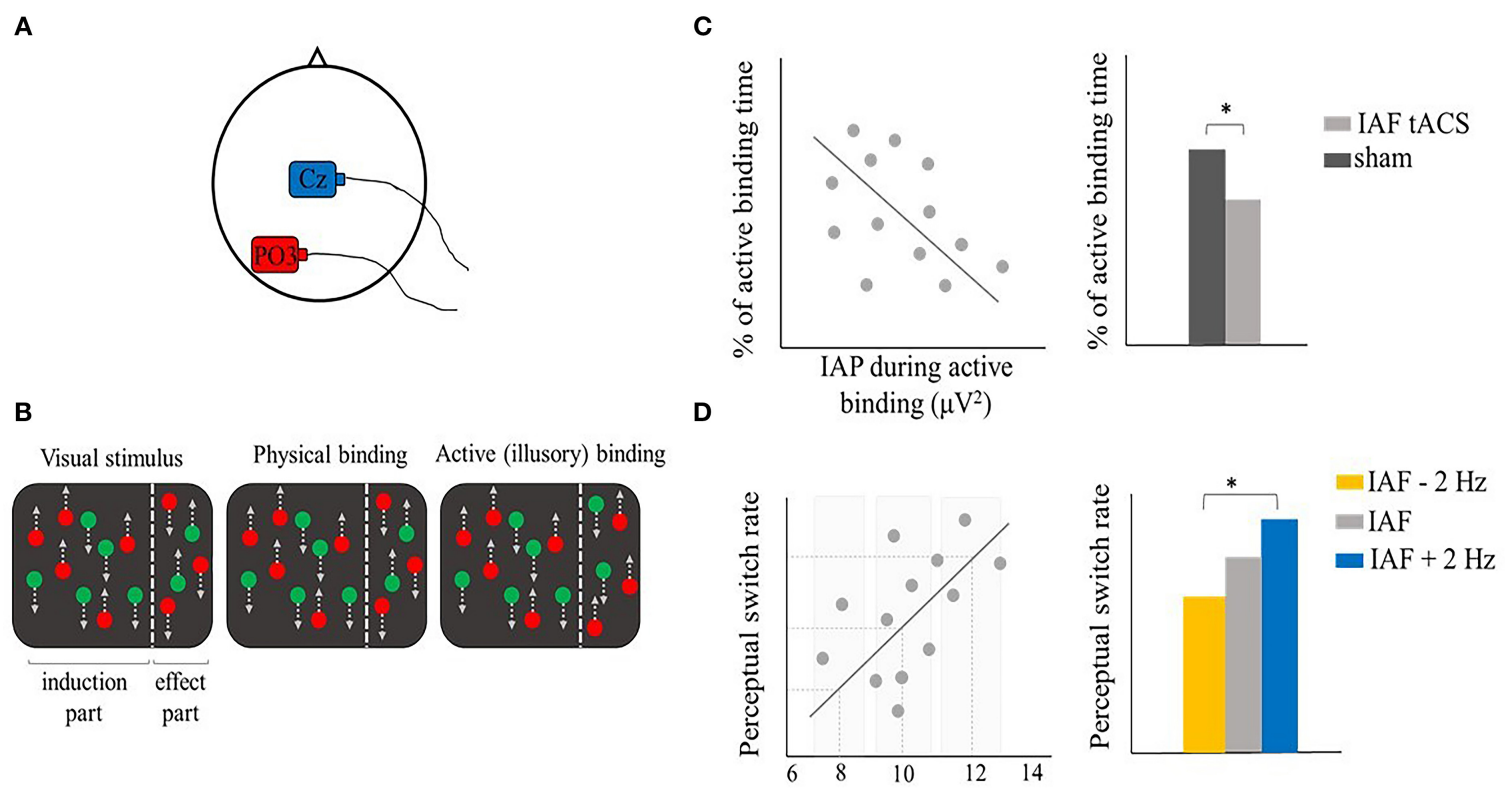
Illustrative representation of the study and results by Zhang et al. (2019). **(A)** tACS montage: the stimulation electrode (red) was placed on PO3, the return electrode (blue) was placed on Cz in the main experiment. **(B)** Illustrative representation of the bistable feature binding stimulus. The visual stimulus was formed by red or green dots that moved either downwards or upwards (as indicated by the gray dotted arrow). The stimulus was divided into an induction part and an effect part, where color and motion were presented in opposite directions (left panel). The stimulus could be either perceived as its physical appearance (central panel) or as active binding, where same-colored dots are perceived moving in the same direction in the induction and effect part (right panel). **(C,D)** Illustrative representation of the main results. **(C)** IAP negatively correlated with the reported active binding time, as shown by EEG results (left panel). IAF tACS significantly reduced the reported active binding time compared to sham, likely by enhancing the alpha power (right panel). **(D)** IAF positively correlated with the perceptual switch rate, as shown by EEG results (right panel). tACS at higher IAF frequencies (e.g., IAF + 2 Hz) increased the perceptual switch rate, likely reducing the perceptual window of physical and/or active binding (right panel). *means that a significant difference was reported.

Oscillatory activity has been also related to feature binding, and neural oscillations are suggested to promote effective communication across different brain areas, allowing the integration of visual features together (Singer and Gray, 1995). Some studies explored the role of gamma oscillations in feature binding (Singer and Gray, 1995; Tallon-Baudry and Bertrand, 1999), which are usually thought to represent local neural processes (Arieli et al., 1995). Zhang et al. (2019) focused instead on the role of alpha activity, starting from evidence that some feature binding problems need the recruitment of several distant brain areas (Koivisto and Silvanto, 2012; Zhang et al., 2014). They used a bistable feature binding stimulus designed by Wu et al. (2004), formed by two groups of dots, one moving downwards and the other upwards. Different moving directions corresponded to different colors (either green or red). The stimulus was divided into a central part (i.e., the induction part) and a right peripheral part (i.e., the effect part) where color and motion were presented in opposite directions (e.g., green downwards moving dots in the central part correspond to green upwards moving dots in the right peripheral part of the stimulus and vice versa). This ambiguous stimulus created two possible color-motion binding percepts that could switch between a “physical” binding—where color and motion of the dots are bound together according to their physical appearance, as previously described—or an “active” (illusory) binding—where color and motion of the dots are erroneously bound together, such that same-colored dots in the central and peripheral parts are perceived moving in the same direction (Figure 6B). Zhang et al. first investigated the relationship between color-motion binding and EEG alpha activity. They first showed a negative correlation between individual alpha power (IAP) and the reported time of active binding perception (Figure 6C, left panel). Starting from this evidence, the authors applied IAF tACS to the left posterior area (electrode position PO3) (Figure 6A), hypothesizing an effect on the active binding time. Indeed, compared to a sham condition, IAF tACS caused a decreased time proportion of the active binding, likely by enhancing IAP, in line with previous research (Figure 6C, right panel) (Zaehle et al., 2010; Helfrich et al., 2014b). Moreover, this effect was specific to the stimulation site (i.e., PO3), since tACS over the right parietal area (electrode position PO4) showed no significant differences compared to sham. Authors also found a positive correlation between the individual alpha frequency (IAF) and the perceptual switch rate, meaning that the higher the IAF, the more frequent was the reported switch rate between the two bistable percepts (Figure 6D, Left panel). As previous studies showed (Cecere et al., 2015; Minami and Amano, 2017), tACS may drive the endogenous oscillatory activity toward lower or higher frequency values than IAF, consequently modulating perceptual performance in different directions (e.g., IAF + 2 Hz or IAF – 2 Hz). Following this logic, in a second experiment, Zhang and colleagues applied tACS at IAF + 2 Hz and IAF – 2 Hz to explore whether the manipulation of alpha frequency could modulate the perceptual switch rate of the bistable stimulus (note, however, that the manipulation of alpha frequency can be only assumed as no EEG activity was recorded during or after tACS). They found that a higher stimulation frequency (i.e., IAF + 2 Hz) was related to a greater perceptual switch rate, likely by reducing the perceptual window of physical and/or active binding (Figure 6D, right panel). Altogether, these results support a causal role of alpha oscillations in feature binding that may be accounted for by their association with interareal interactions and long-range neural communications that have been shown to be crucial in some types of feature bindings (like the active binding in the study by Zhang et al.) (Von Stein and Sarnthein, 2000; van Driel et al., 2014).

Overall, the studies summarized above provide suggestive evidence of the causal role of brain oscillations in spatial and feature binding (see Table 1 for a summary of these studies). tACS has been proven effective in manipulating oscillatory activity which has been correlated to contour integration (Stonkus et al., 2016), perceptual segregation within crowded visual scenes (Battaglini et al., 2020a), and feature binding (Zhang et al., 2019). In particular, Stonkus et al. (2016) provided preliminary evidence of the causal role of theta oscillations in contour binding by applying in-phase tACS over occipital and parietal regions right before stimulus presentation. Beta activity seems to be causally involved in resolving correct target segregation in a crowded condition (Battaglini et al., 2020a), possibly by promoting a switch from global (crowded) to local (uncrowded) processing. Finally, some forms of feature binding may be mediated by long-range interareal communication, likely mediated and causally supported by alpha activity, as shown by Zhang et al. (2019). Even if these studies highlighted some potential limitations (e.g., partial results, no tACS phase manipulation, fixed stimulation frequency), it is worth noting that only recently tACS started to be used as a tool to manipulate complex aspects of visual and cognitive processes.

## Spatio-Temporal Binding

**Figure 7:**
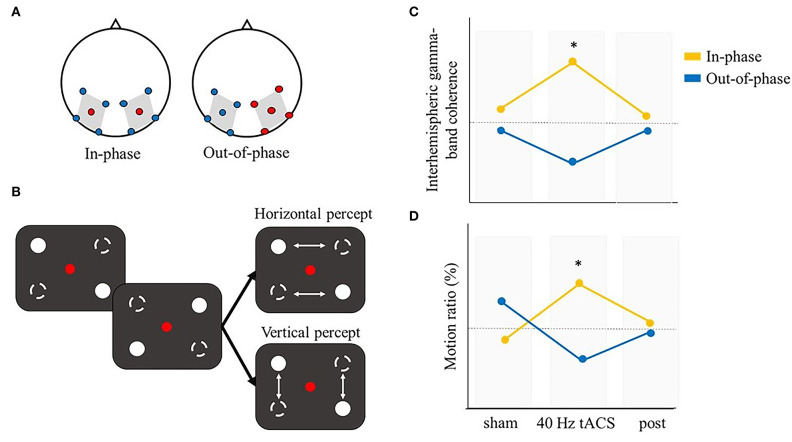
Illustrative representation of the study and results by Helfrich et al. (2014a). **(A)** mc-tACS montage: in the in-phase condition (left panel) electrodes were positioned to optimally stimulate right and left occipital-parietal regions. In the out-of-phase condition (right panel) electrodes were positioned according to Strüber et al. (2014), covering positions P7-PO7 and P8-PO8. **(B)** Schematic representation of the stroboscopic alternative motion (SAM) task: participants could alternatively perceive either a horizontal or vertical percept. **(C,D)** Illustrative representation of the main results. **(C)** In-phase (out-of-phase) interhemispheric gamma tACS effectively increased (decreased) interhemispheric gamma-band coherence. **(D)** The motion ratio (i.e., time_horizonal_/time_total_ ) is a measure of the mean duration of horizontal motion perception. During 40 Hz tACS, in-phase stimulation significantly increased the perceived motion ratio compared to sham or post-stimulation. *means that a significant difference was reported.

So far, we looked at the binding process separately within the spatial and temporal domain. However, many experimental paradigms are difficult to classify into one of these two general categories. The stroboscopic alternative motion (SAM) seems a particularly good example of such paradigms. The SAM is a type of bistable perception where two pairs of LEDs moving on opposite diagonals of a square could be integrated and perceived differently, giving rise to distinct phenomenological experiences, namely a horizontal or vertical apparent motion (Glaser and Chaudhuri, 1991, Strüber et al., 2014) (Figure 7B). Critically, the switch between the two percepts has been suggested to depend on the level of interhemispheric gamma-band coherence. In particular, higher interhemispheric gamma-band coherence would be required for the stimulus to be integrated into a horizontal motion as compared to a vertical motion (Rose and Büchel, 2005). In this case, gamma synchronization would play a role in the resolution of the binding problem during ambiguous stimulation (Engel and Singer, 2001; Engel et al., 2001; Doesburg et al., 2005). Some studies further explored the potential causal role of this oscillatory correlate. Strüber et al. used a classic SAM paradigm in order to assess the role of interhemispheric gamma-band coherence in ambiguous motion perception (Strüber et al., 2014). They applied 6 or 40 Hz tACS over parietal-occipital regions, targeting the human motion complex (hMT) (electrodes position P7-PO7 and P8-PO8; experiment 1 and 2), or over occipital regions (electrodes position: O1 and O2; experiment 3). tACS over one hemisphere was either out-of-phase (experiment 1 and 2) or in-phase (experiment 3) compared to tACS over the opposite hemisphere. Through this montage, they aimed to, respectively, disrupt or enhance interhemispheric coupling during the task. They found a specific effect of out-of-phase 40 Hz tACS stimulation in biasing bistable apparent motion causing an increased interhemispheric gamma coherence between pre- and post-stimulation, coupled with a decreased proportion of perceived horizontal motion (i.e., an increased proportion of perceived vertical motion). Even if the out-of-phase stimulation was expected to cause a decreased interhemispheric gamma synchronization, this finding still supports the involvement of interhemispheric gamma-band coherence in bistable motion perception and importantly, it shows that tACS modulates interhemispheric synchronized oscillatory activity in a frequency-specific manner. A subsequent study (Helfrich et al., 2014a) adopted a similar setup, while using mc-tACS, that allowed for a more focused stimulation by employing 5 Ag/AgCl electrodes (Dmochowski et al., 2011; Kuo et al., 2013). In the out-of-phase condition, electrodes were placed on the same regions of experiment 1 and 2 in the study by Strüber et al. (2014) (i.e., parietal-occipital regions, optimally targeting hMT), while in the in-phase stimulation they opted for a more lateral stimulation, targeting the extrastriate visual cortex (Figure 7A). Through EEG co-registration, they first confirmed previous findings that tACS modulates interhemispheric oscillatory coupling within the gamma band. Importantly, they corroborated the results by Strüber et al. of an out-of-phase 40 Hz tACS effect biasing the proportion of vertical motion percepts. Moreover, in line with the literature, they also showed that in-phase stimulation promoted the perception of horizontal motion, likely by increasing gamma interhemispheric synchrony (Figures 7C,D). However, no gamma power changes were reported. Instead, a prominent alpha power reduction during 40 Hz tACS was observed over lateral parieto-occipital regions. The increased gamma synchronization coupled with a decreased alpha power found in this study is in line with the hypothesis of an antagonistic role of alpha and gamma oscillations in the parieto-occipital region (Jensen et al., 2012). Further analyses showed that this result is likely due to a cross-frequency coupling (CFC), which would be influenced by the external 40 Hz stimulation (Helfrich et al., 2014a).

Both studies (Helfrich et al., 2014a; Strüber et al., 2014) successfully demonstrated that tACS is effective in shaping interhemispheric synchrony, as shown by their EEG results. However, while Strüber et al. did not find any effect during the in-phase stimulation, Helfrich et al. found a different behavioral effect on the perceived motion after both out-of-phase and in-phase tACS, in line with the literature (Rose and Büchel, 2005). Helfrich et al. (2014a) suggest that the negative finding of Strüber et al. is likely due to a different current distribution in the in-phase condition between the two studies. By investigating the electric field simulation, it is evident that while in both studies the out-of-phase stimulation targeted extrastriate regions (leading to the expected behavioral results), the in-phase stimulation engaged different regions: extrastriate areas in Helfrich et al. (2014a) and the occipital poles in Strüber et al. (2014). This may explain the absence of a behavioral effect in the latter study and highlights the importance of carefully evaluating the electric field distribution and electrode positioning in order to increase tACS effectiveness and reproducibility.

Another study investigated the role of gamma oscillations in bistable perception, employing a structure-from-motion stimulus (Cabral-Calderin et al., 2015). This stimulus is formed by two fields of moving dots that are integrated into a rotating sphere that could be alternatively perceived moving rightwards or leftwards, creating an ambiguous stimulation. It has been suggested that the switch from one percept to the other is likely dependent on synchronous oscillatory activity between frontoparietal and extrastriate regions (Sterzer et al., 2002; Sandberg et al., 2014). In particular, several studies showed that perceptual switches are linked to decreased alpha activity (Strüber and Herrmann, 2002; Mathes et al., 2010) and an increased gamma power and synchrony, such that destabilization of one percept may be mediated by alpha oscillations, while gamma activity would resolve the binding of the ambiguous stimulation into the new percept (Strüber et al., 2000; Doesburg et al., 2005). Cabral-Calderin et al. (2015) tested a causal role of these oscillatory correlates in perceptual switches by using 60, 80 Hz tACS (i.e., low or high gamma tACS) and 10 Hz tACS (i.e., alpha tACS) over the occipital cortex (electrode position Oz). They showed that spontaneous perceptual switches were increased by 60 Hz tACS (i.e., low gamma tACS). The authors speculate that gamma tACS may disturb motion-related visual regions, disrupting the emergence of a coherent motion percept that would trigger parietal regions to reinterpret the stimulus, leading to increased perceptual reversals. However, as no EEG results were reported, we cannot be sure of an effective modulation of gamma activity after 60 Hz tACS. Finally, even if correlational studies showed a possible role of alpha activity in perceptual switch rates (Strüber and Herrmann, 2002; Mathes et al., 2010), this study found a causal link of low gamma activity only, highlighting the advantage of tACS in identifying brain oscillations that are causally relevant for binding processes during ambiguous stimulation. However, it should be noted that the lack of evidence of a role of alpha activity may be also due to methodological limitations (e.g., fixed stimulation frequency) and it does not rule out the possibility of a causal role of the alpha rhythm in binding mechanisms under ambiguous stimulation.

Kar and Krekelberg showed that alpha activity may be causally related to motion perception. In that study, spatio-temporal binding was measured with a motion discrimination sensitivity task, in which participants were asked to report the motion direction of a group of moving dots (random dot kinematogram; RDK) (Kar and Krekelberg, 2014). The authors showed that 10Hz tACS applied over hMT (electrode position covering PO7-PO3) contralateral to the stimulus improved motion discrimination performance. In a second experiment, the authors tested the tACS effect on the motion aftereffect (i.e., the illusion of motion in the opposite direction of a moving adapter when presenting a stationary or random-moving stimulus) by applying the stimulation before the test stimulus, when participants were shown with an adapter (i.e., a RDK with dots moving upwards with 100% coherence). Results showed a reduction of motion aftereffect when 10 Hz tACS was applied contralaterally during adaptation. Together with additional experiments that found no tACS effects when stimulation was applied in a prestimulus period or right after adaptation, this is consistent with the idea that improvement of motion sensitivity during 10Hz tACS is likely due to its effect on motion adaptation.

Overall, the studies here described are in line with the idea of a causal role of gamma activity in integrating ambiguous visual information in the perceived stimulus and in the switch from one percept to the other. Notably, they provided evidence of tACS as a promising technique not only to modulate the power or frequency of brain oscillations but also to selectively interfere with interhemispheric synchronization, relevant for various binding processes, as previously shown (Rose and Büchel, 2005; Costa et al., 2017). The alpha rhythm may be also implicated in spatio-temporal binding, as highlighted by the effect of 10 Hz tACS on motion adaptation (Kar and Krekelberg, 2014). Interestingly, gamma (i.e., 40 Hz) tACS during a spatio-temporal binding task was shown to affect gamma synchronization as well as alpha power, suggesting a possible role of alpha-gamma CFC in resolving binding across both space and time.

## Clinical Implications of tACS-Induced Changes in Perception

Reproducible and predictable tACS effects on binding processes in healthy populations may lie the foundation for a possible application of this technique as a rehabilitation tool with clinical populations where these processes and their oscillatory correlates were shown to be altered. Several conditions, like autism spectrum disorder (ASD), developmental dyslexia, and schizophrenia have been associated with abnormalities in perceptual binding (Dakin and Frith, 2005; Silverstein and Keane, 2011; Gori et al., 2016; Zvyagintsev et al., 2017; Zhou et al., 2018; Stein, 2019) that have been linked to alterations in the oscillatory dynamics in ASD (Uhlhaas and Singer, 2012; Simon and Wallace, 2016; Ronconi et al., 2020b), developmental dyslexia (Vidyasagar, 2019), and schizophrenia (Moran and Hong, 2011; Uhlhaas and Singer, 2012; Başar-Eroglu et al., 2016; Mathes et al., 2016; Rürup et al., 2020). First, it should be evaluated whether these alterations in the oscillatory dynamics may play a causal role in perceptual integration abnormalities in these pathologies. Secondly, future research should attempt to ameliorate disrupted binding processes by directly manipulating relevant brain oscillations. Both these goals require a tool that is able to shape the rhythmic activity of the brain. Throughout this review, we provided evidence that tACS may be suitable for this goal.

Even if some methodological challenges need to be addressed by future studies, some research has already shown a successful tACS effect in the treatment of schizophrenia (Ahn et al., 2019; Haller et al., 2020; for a review see Elyamany et al., 2020). These studies showed that tACS is effective in modulating key aspects of schizophrenia, such as working memory performance (Sreeraj et al., 2017), negative symptoms (Kallel et al., 2016; Haller et al., 2020) and auditory hallucinations (Mellin et al., 2018; Ahn et al., 2019). Sreeraj et al. (2017) limited their investigation on the online effects of theta vs. gamma tACS, applied for 20 min over the left dorsolateral prefrontal cortex (DLPFC, electrode position F3) and left posterior parietal region (electrode position P3). With a randomized trial, Mellin et al. (2018) applied 20 min 10 Hz tACS twice a day for 5 consecutive days over left frontal and temporo-parietal areas and found no tACS benefits on auditory hallucinations past one week follow-up. Kallel et al. (2016) and Haller et al. (2020) applied tACS over the left and right DLPFC (electrodes position F3-F4) for an extensive period of time (20 sessions of 20 min once a day and 20 sessions of 10 min twice a day, respectively) but did not report any follow-up. All in all, the results from these case studies should be taken with caution due to a small sample size, the absence of a control group or a sham condition and the lack of a double-blind procedure. Critically, in these studies no EEG was recorded during or after stimulation, thus no conclusion on the physiological effects of tACS can be drawn.

More convincing evidence of a potential role of tACS as a rehabilitation tool in schizophrenia was provided by Ahn et al. (2019). Employing a randomized, double-blind sham-controlled clinical trial, they found that 10 Hz tACS applied over DLPFC (electrodes position F3-Fp1) and the temporo-parietal junction (electrodes position T3-P3) not only enhanced alpha oscillations, but also 40 Hz auditory steady-state responses (ASSR), previously found to be reduced in patients with schizophrenia (Brenner et al., 2009). More importantly, they also reported that the enhancement of alpha oscillations and 40 Hz-ASSR was accompanied by a reduction in auditory hallucinations, with some participants showing this effect up to 1-months later. Schizophrenia is also associated with altered neural activity and impaired spatio-temporal integration as shown by patients' performance during a stroboscopic alternative motion (SAM) task (Başar-Eroglu et al., 2016; Mathes et al., 2016; Rürup et al., 2020). However, no tACS studies to date tried to modulate spatio-temporal binding processes in this pathology. Interestingly, ambiguous perception in a SAM task was successfully modulated by interhemispheric gamma tACS in a non-clinical sample (Helfrich et al., 2014a), suggesting a possible application of this technique in patients with schizophrenia. Altered perceptual binding and oscillatory dynamics were also found in ASD (Dakin and Frith, 2005; Uhlhaas and Singer, 2012; Simon and Wallace, 2016; Zhou et al., 2018; Ronconi et al., 2020b). Several studies applied transcranial direct current stimulation as a possible rehabilitation tool for people with ASD (for recent reviews see Osório and Brunoni, 2019; Khaleghi et al., 2020). Nonetheless, tACS application in the exploration of ASD and its oscillatory dynamics is still lacking, and future research is needed to establish tACS as a possible rehabilitation tool for ASD. Notably, ASD has been related to altered horizontal binding when presented with a metastable motion quartet, a stimulus similar to the one employed in a SAM task. This result may be linked to decreased cross-hemispheric communication (David et al., 2010). Interestingly, horizontal motion perception may be promoted by in-phase 40 Hz tACS (Helfrich et al., 2014a), suggesting a possible future direction of tACS as a rehabilitation tool for ASD.

## Current Limitations of tACS Studies Exploring Perceptual Binding

Some of the studies reviewed above failed in showing a robust tACS effect in binding processes (Strüber et al., 2014; Stonkus et al., 2016; Battaglini et al., 2020b; Ronconi et al., 2020a). These negative or partial findings are likely related to methodological differences that should prompt future research on how we can improve tACS efficacy by modeling some parameters, such as inter-individual variations of electrical field distribution across the scalp, position and number of electrodes (i.e., two-channel tACS vs. mc-tACS), and individualized intensity and frequency of stimulation. Altogether, the modeling of these parameters would take into account individual differences in skull thickness, skin impedance, and in the participant's individual peak frequency of brain oscillations in order to reduce the variability of effects shown in neurostimulation studies (Ali et al., 2013; Wagner et al., 2014; Bikson et al., 2018; Evans et al., 2020). Reliable and reproducible tACS studies should also consider other important issues that are worth mentioning. First, most studies employing non-invasive brain stimulation have been conducted with relatively low sample sizes (Guerra et al., 2020). Sham blinding should also be considered when judging the research quality. Indeed, some studies raised some concerns on the reliability of currently used blinding procedures (Horvath, 2015; Fonteneau et al., 2019). Some protocols have been developed in order to improve sham efficacy. One is to use multi-channel montages that have the advantage of recreating skin sensations while keeping the cortical electrical field near zero. Another proposed solution may be the use of topical pre-treatments both in the sham and active group in order to reduce tingling and itching sensations often experienced during stimulation (Fonteneau et al., 2019). Finally, brain stimulation research reliability may also benefit from study pre-registration and direct replications of published results, in line with recent attempts to improve research quality in several fields of psychology and neuroscience.

## Conclusions and Future Directions

The goal of this review was to provide a comprehensive picture of current evidence regarding the potential causal role of brain oscillations in binding processes, both in the temporal and spatial domain, in light of previous neurophysiological research that linked several binding mechanisms to precise oscillatory correlates (e.g., Varela et al., 1981; Rose and Büchel, 2005; Busch and VanRullen, 2010; Jensen et al., 2012; Hanslmayr et al., 2013; McLelland et al., 2016; Wutz et al., 2016, 2018; for reviews see VanRullen, 2016; Costa et al., 2017; Ronconi et al., 2017; White, 2018). However, these studies did not directly manipulate oscillatory activity, meaning that no causal relationship can be inferred (Herrmann et al., 2016; Bergmann and Hartwigsen, 2020). tACS has been recently shown to effectively modulate brain oscillations in a frequency-dependent manner (for reviews see Herrmann et al., 2016; Cabral-Calderin and Wilke, 2020), allowing for a direct investigation of their causal involvement in binding processes.

Overall, the studies reviewed here provided converging evidence that tACS is effective in modulating brain oscillations, previously related to temporal and spatial binding processes (Helfrich et al., 2014a; Strüber et al., 2014; Cecere et al., 2015; Minami and Amano, 2017; Zhang et al., 2019; Battaglini et al., 2020a). In general, endogenous rhythmic activity cannot be merely considered as an epiphenomenon, but as a mechanism that actively supports binding mechanisms across space and time (and potentially features), allowing for the integration of different information into a unified and meaningful percept.

In particular, current evidence suggests a relationship between temporal binding and occipital alpha activity that seems to dictate the sampling frequency of sensory information, creating discrete temporal windows of integration and segregation, as previously reported by electrophysiological studies (Busch and VanRullen, 2010; Jensen et al., 2012; McLelland et al., 2016; Wutz et al., 2016, 2018; Ronconi et al., 2017). Notably, some studies showed a direct modulation of these discrete temporal windows by alpha tACS, both at a fixed stimulation frequency (e.g., 10 Hz) (Battaglini et al., 2020b) or at individually tailored frequencies (Cecere et al., 2015; Minami and Amano, 2017). Although some studies recently showed that the detection of near-threshold stimuli was modulated by the phase of tACS when applied at alpha frequency (Helfrich et al., 2014b; de Graaf et al., 2020), there is no convincing evidence to date that tACS phase is able to shape temporal integration processes (Ronconi et al., 2020a). Nevertheless, it is worth mentioning that tACS studies thus far mainly focused their investigation on the role of alpha frequency, especially within the temporal domain, building a useful, yet incomplete, picture of the causal role of different rhythms (e.g., theta rhythm) in modulating the temporal resolution of perception at different temporal scales (Busch et al., 2009; Ronconi et al., 2017). Future studies may take advantage of the current correlational evidence in order to explore how temporal binding is modulated by multiple brain rhythms (see Table 2).

**Table 2:** Some future research questions that may be addressed by future studies interested both in fundamental and clinical aspects of visual binding processes.

**Future research questions**
•M/EEG studies showed a correlation between alpha/theta frequency and temporal binding. However, tACS studies mainly focused on alpha activity, leaving the potential causal role of theta activity largely unexplored. Future research may attempt to investigate whether and in which conditions theta activity causally supports temporal binding •The identification of several types of binding processes (e.g., temporal and spatial) may be somehow artificial. Are different types of binding part of a more general binding mechanism? •How different brain oscillations and brain regions interact in order to achieve a correct binding? Can cross-frequency coupling and long-range synchronization be potential neural mechanisms underlying binding? •In real-life situations, sensory information is much more complex than in the lab. Does our brain solve the binding problem in similar ways, or does it rely on different processing when dealing with more realistic and dynamic situations? •Several disorders (e.g., schizophrenia and ASD) showed impaired binding processes and altered oscillatory dynamics. Is tACS a valuable tool for a potential intervention on these disorders?

When looking at tACS evidence on spatial binding, the emerging picture is consistent with a role of theta and beta oscillations in resolving correct perceptual integration/segregation across space. In particular, theta phase has been associated with transient windows of information transfer between low-level and high-level visual regions (Hanslmayr et al., 2013) that would be required to represent local visual features and correctly integrate them according to their spatial location (Roelfsema, 2006). The causal involvement of this rhythm has been partially supported by Stonkus et al. (2016) that found a sinusoidal modulation of task performance when synchronizing occipital and parietal regions when in-phase theta tACS was applied. Beta activity has been also shown to be causally involved in correct target feature integration in a crowding condition, in agreement with previous EEG evidence (Ronconi et al., 2016b; Ronconi and Bellacosa Marotti, 2017), likely by promoting a dorsal-to-ventral feedback that would allow an efficient extraction of information in cluttered visual scenes (Battaglini et al., 2020a).

It would be interesting in future work to explore how and whether different types of binding may interact in order to achieve a unified representation in space and time. Indeed, we reported some studies investigating perceptual processes involving spatio-temporal binding of ambiguous stimulation and their relation with interhemispheric gamma synchronization (Helfrich et al., 2014a; Strüber et al., 2014). Moreover, Helfrich et al. (2014a) also reported that an increased gamma synchronization during 40Hz tACS was coupled with a decreased alpha power, suggesting a role of CFC in resolving the binding problem. Interestingly, along with interhemispheric gamma synchronization, gamma-theta CFC has been previously suggested as a mechanism involved in horizontal motion perception in a SAM task (Alipour et al., 2016). In line with these findings, it has been proposed that while fast oscillations (i.e., gamma) would synchronize neural activity locally, slow oscillations (e.g., within the theta-alpha range) may play a role when long-range network synchronization is required (e.g., during interhemispheric communication) (Von Stein and Sarnthein, 2000). It should be noted, however, that current tACS studies investigating the role of brain oscillations in binding processes would be not sufficient to draw conclusions about a general binding model and its neural mechanism. Indeed, while the reviewed studies provide initial evidence for a causal relationship between binding processes and their potential oscillatory correlates, the modeling of this relationship would require a more extensive body of evidence coming from both brain stimulation and electrophysiological research. However, it is worth specifying that the distinction between different binding processes in the present review was made to create a clearer, yet somehow artificial, separation of what we think is a more complex and comprehensive binding mechanism. In fact, when looking at real-life situations, we constantly need to bind together information at different timescales and across different locations in space.

Most of the studies reviewed above used simple tasks (e.g., bind together two flashes in time or spatially integrate a contour presented in a gray background), so it is not surprising to find a unique putative oscillatory correlate linked with a simple perceptual process. We may speculate that when considering more complex tasks, more than one frequency may be relevant at the same time. For example, it has been shown that during perception, events are organized through a hierarchy of timescales. Indeed, perceptual stimuli are thought to be integrated along increasingly prolonged timescales, such that each brain region would segment sensory information at its favorite timing, starting with short segmentation periods in low-level sensory cortices (e.g., segmenting two stationary flashes in early visual cortex) and longer periods in high-level regions (e.g., integrating complex stimuli moving in space) (Baldassano et al., 2017). This increase in temporal window may mirror the increase in spatial receptive field sizes in higher areas of the visual processing hierarchy. It might be speculated that different timescales may correspond to different oscillatory dynamics, with short timescales corresponding to higher frequencies and longer timescales with lower frequencies. Support for this view has been provided by Ronconi et al., demonstrating that the perceptual sampling of visual events across different timescales depends on multiple EEG oscillatory rhythms (Ronconi et al., 2017). In sum, the exploration of a possible interplay between several binding processes and their oscillatory correlates would lead to new and fascinating insights, with potential implications for our basic understanding of neural dynamics and possible clinical applications to neurological disorders. Future research may further explore how different binding processes (e.g., temporal and spatial) interact in order to achieve a general binding mechanism that our brain may adopt in more dynamic and complex situations (see Table 2).

## Author Contributions

AG: conceptualization, literature search, writing—original draft, and visualization. MM, LB, and DM: conceptualization and writing—reviewing and editing. LR: conceptualization, writing—reviewing and editing, literature search, and supervision. All authors contributed to the article and approved the submitted version.

## Conflict of Interest

The authors declare that the research was conducted in the absence of any commercial or financial relationships that could be construed as a potential conflict of interest.
